# Neuroanatomical correlates of gross manual dexterity in children with unilateral spastic cerebral palsy

**DOI:** 10.3389/fnhum.2024.1370561

**Published:** 2024-04-09

**Authors:** Elena Beani, Veronica Barzacchi, Elena Scaffei, Beatrice Ceragioli, Fabrizia Festante, Silvia Filogna, Giovanni Cioni, Simona Fiori, Giuseppina Sgandurra

**Affiliations:** ^1^Department of Developmental Neuroscience, Istituto di Ricovero e Cura a Carattere Scientifico (IRCCS) Fondazione Stella Maris, Pisa, Italy; ^2^Department of Clinical and Experimental Medicine, University of Pisa, Pisa, Italy; ^3^Tuscany Ph.D. Programme of Neuroscience, University of Florence, Florence, Italy

**Keywords:** children, unilateral cerebral palsy, gross manual dexterity, neuroanatomical correlates, mirror movements, Magnetic Resonance Imaging

## Abstract

Unilateral spastic Cerebral Palsy (UCP) results from congenital brain injury, and Magnetic Resonance Imaging (MRI) has a role in understanding the etiology and severity of brain insult. In UCP, functional impairment predominantly occurs in the upper limb (UL) of the more affected side, where manual ability and dexterity are typically reduced. Also, mirror movements (MMs), are often present in UCP, with a further possible negative functional impact. This study aims to investigate the relationships among neuroanatomical characteristics of brain injury at MRI, manual functional impairment and MMs, in children with UCP. Thirty-five children with UCP participated in the study (20, M = 15, F, mean age 9.2 ± 3.5 years). Brain lesions at MRI were categorized according to the Magnetic Resonance Classification System (MRICS) and by using a semi-quantitative MRI (sqMRI) scale. Gross manual performance was assessed through Manual Ability Classification System (MACS) and the Box and Block Test (BBT), and MMs by Woods and Teuber scale, for both hands. Non-parametric correlation analyses were run to determine the relationship between neuroanatomical and functional features. Regression models were run to explore the contribution of neuroanatomical features and MMs to UL function. Correlation analyses revealed moderate to strong associations between sqMRI scores contralateral to the more affected side and UL functional impairment on MACS and BBT, with more severe brain injuries significantly correlating with poorer function in the more affected hand. No association emerged between brain lesion severity scores and MMs. MRICS showed no association with MACS or BBT, while a significant correlation emerged between MRICS category and MMs in the more affected hand, with brain lesion category that are suggestive of presumed earlier injury being associated with more severe MMs. Finally, exploratory regression analyses showed that neuroanatomical characteristics of brain injury and MMs contributed to the variability of UL functional impairment. This study contributes to the understanding of the neuroanatomical and neurological correlates of some aspects of manual functional impairment in UCP by using a simple clinical brain MRI assessment.

## 1 Introduction

Unilateral spastic Cerebral Palsy (UCP) is the most common Cerebral Palsy (CP) subtype, reported in around 25% of children with CP (Yates, 2014). UCP is due to congenital or early acquired brain injury in the developing brain, with Magnetic Resonance Imaging (MRI) supporting the understanding of the etiology of brain insult (Ashwal et al., 2004). MRI abnormalities in UCP, as in other forms of CP, are commonly categorized according to the timing of injury, from the early gestational to perinatal period, in brain maldevelopments, predominant white matter injury, predominant gray matter injury or miscellaneous (Cioni et al., 1999; Himmelmann et al., 2017). The clinical phenotype in UCP is unilateral by definition, with spasticity being the prominent motor feature, due to the involvement of the corticospinal tract (CST) for voluntary motor control in the brain. Approximately one third of UCP children have bilateral asymmetrical brain injury on MRI (Cioni et al., 1999; Holmefur et al., 2013; Scheck et al., 2016), which recurs more often in predominant white matter injury (Cioni et al., 1999). In UCP, motor impairment mostly involves one side of the body with up to 30% of subjects showing a certain degree of impairment in the presumed unaffected side (Cioni et al., 1999; Arnould et al., 2014), which may be at least in part due to the abovementioned bilateral lesion distribution.

Functional impairment in the upper limb (UL) is a prevalent challenge among children diagnosed with UCP. The Manual Ability Classification System (MACS) reliably assesses manual performance in everyday activities in children with UCP. However, while MACS aligns with the performance aspect of the International Classification of Functioning, Disability, and Health (Leonardi et al., 2022), it does not assess maximal capacity and is not designed to differentiate between the capabilities of the two hands in UCP (Eliasson et al., 2006). Indeed, children with UCP often exhibit a preference for the use of the less affected hand over their more affected one. Hand functional impairment potentially impedes motor skill development, uni- and bimanual performances and hinders engagement in daily activities (Woods and Teuber, 1978; Wallen and Stewart, 2016), with manual dexterity being a strong predictor of UL functionality in daily life activities (Alt Murphy et al., 2015). In addressing these constraints, clinicians invest significant effort and resources to describe UL functional impairment. In particular, the Box and Block Test (BBT) serves to evaluate gross manual dexterity (Mathiowetz et al., 1985; Bleyenheuft et al., 2015; Decraene et al., 2021; Liang et al., 2021). This assessment tool is user-friendly, standardized for clinical use, easily accessible, straightforward to administer, and it does not require specialized settings to be performed (Goodkin et al., 1988; Lin et al., 2010). BBT is widely adopted as an outcome measure in adults, and it has recently established reliability and validity specifically in UCP (Decraene et al., 2021; Liang et al., 2021).

It has been shown that presumed category of the brain insult has a certain association with functional impairment in UCP, since hand function is more severely impaired in perinatal gray matter injury compared to earlier lesions (Cioni et al., 1999). Also, brain lesion characteristics in the hemisphere contralateral to more affected hand, assessed by a semi-quantitative procedure (sqMRI) applied to clinical MRI, appeared to be associated with measures of uni- and bimanual abilities (Fiori et al., 2015). Among factors that may impact on functional impairment in UCP, plasticity of the CST for voluntary motor control has a well-established role (Staudt, 2010; Fiori et al., 2018). Findings from animal and human models of congenital brain injury, indicate that voluntary motor control of the more affected hand can shift in the contralesional motor cortex, with a negative impact on motor function (Eyre et al., 2001; Holmström et al., 2010; Staudt, 2010). In this condition, motor commands originating from the contralesional hemisphere can result in the simultaneous activation of both hands, as demonstrated by single-pulse transcranial magnetic stimulation (TMS) (Carr, 1996; Eyre, 2007). The neurological correlates of this phenomenon are persistent Mirror Movements (MMs), which appear to be of value to estimate motor system developmental plasticity in UCP (Riddell et al., 2019). MMs are defined as ‘involuntary movements of a bodily segment that replicate the intentional movement of the corresponding homologous segment on the opposite side’ (Woods and Teuber, 1978), and predominantly manifest in the UL. Physiological MMs emerge in newborns, exhibit a marked decline between 5 and 8 years, and vanish after the age of 10 (Koerte et al., 2010). MMs are likely a result of physiologically incomplete interhemispheric inhibition by corpus callosum during unilateral tasks, which trigger activation of both motor cortices (Cincotta and Ziemann, 2008). MMs are frequently present in UCP, predominantly in the less affected hand, with their etiology remaining not fully comprehended (Riddell et al., 2019; Magne et al., 2021). In UCP, MMs are related at least in part to pathologically acquired incomplete transcallosal inhibition, but also to the abovementioned persistence of ipsilateral CST projections between the unaffected motor cortex and the affected hand as the result of congenital brain injury (Kuhtz-Buschbeck et al., 2000; Riddell et al., 2019). CST reorganization in children with UCP may rely on both timing and extent of brain lesion. However, the precise relationship between lesion characteristics and MMs is yet to be explored, as well as the impact of MMs manifestation on manual abilities in UCP.

The aim of the current study was to investigate the relationships among neuroanatomical characteristics of brain injury (MRICS category and severity at clinical MRI), functional impairment of gross manual dexterity (Box and Block Test, BBT), and MMs, in a cohort of subjects with UCP. Furthermore, we explored the contribution of neuroanatomical features and MMs to the variability of functional impairment at BBT and MACS. We hypothesized that a more severe brain lesion in the hemisphere contralateral to more affected side correlated with reduced manual dexterity, and that brain lesion characteristics and MMs have an impact on variability of functional impairment.

## 2 Materials and methods

### 2.1 Participants

Children and adolescents were enrolled in this single-center study according to the following inclusion criteria: (i) confirmed diagnosis of UCP; (ii) age between 4 and 18 years; (iii) availability of a full set of clinical MR images acquired after 3 years of age; (iv) cognitive level within normal limits (IQ ≥70), assessed before recruitment on the Wechsler Preschool and Primary Scale of Intelligence-Third Edition (WPPSI-III) (Warschausky, 2011), Wechsler Intelligence Scale for Children Fourth Edition (WISC-IV) (Grizzle, 2011) or Wechsler Adult Intelligence Scale (WAIS) (Wechsler, 2019) to allow for full collaboration during clinical testing. Exclusion criteria were: (i) intramuscular botulinum toxin A (BoNT-A) injection or (ii) orthopedic surgery, both within 6 months prior to enrolment; (iii) intensive periods of intervention (daily interventions for at least 3 weeks) for UL at any age prior to enrolment; (iv) presence of behavioral comorbidities that preclude adequate cooperation in clinical assessment. Recruitment was carried out at IRCCS Stella Maris Foundation, Pisa, Italy from March 2022 to March 2023.

This study was approved by the Pediatric Ethics Committee of Tuscany (53/2022) and parental written informed consent was obtained for all children prior to participation in the study.

### 2.2 Neuroimaging assessment

MRI data were acquired by using a 1.5 T or 3 T MRI scanner (Signa Horizon 1.5; GE, Milwaukee, WI and Premier 3 T, GE, Milwaukee, WI) at IRCCS Stella Maris Foundation for all subjects. Children were all older than 3 years of age at the time of MRI data acquisition. Clinical acquisition protocol includes planar T1, T2 and GRE weighted images, 3D T1, FLAIR and SWI.

MR images were retrospectively collected and assessed with a reliable and valid semi-quantitative scoring system (Fiori et al., 2015) by an experienced child neurologist (SF) and revised by a child neuroradiologist (RP).

#### 2.2.1 Brain lesion MRI assessment

Brain lesion severity at clinical MRI was assessed through a valid and reliable semi-quantitative (sqMRI) scoring system (Fiori et al., 2015). Briefly, the sqMRI scoring procedure is based on a six-axial-slices template with an anatomical correspondence identified with appropriate MRI slices. In summary, all hemispheric and subcortical structure involvement is assessed systematically, thus resulting in a number of subscores and scores. The Hemispheric Score (HS, range: 0–12) results from the sum of the four (frontal, parietal, temporal, occipital) lobar scores on each (right and left) side. The basal-ganglia region score (BGrS, range: 0–9) is the result of subcortical structures involvement and comprises the assessment of basal ganglia (caudate, putamen and globus pallidus) as well as adjacent structures including the posterior limb of internal capsule, thalamus and brainstem on each side. The cerebellum score and the corpus callosum score (CCS) are assessed separately. The sum of all the scores, including cerebellum score and CCS, results in a global score (GS) (range: 0–48). For all scores, higher scores correspond to more severe lesion. For details on the scoring procedure see Fiori et al. (2014, 2015) and Laporta-Hoyos et al. (2018).

Brain MR images were also classified by using the Surveillance of Cerebral Palsy in Europe (SCPE) classification system according to the Magnetic Resonance Imaging Classification System (MRICS) (Himmelmann et al., 2017). The MRICS identifies four main categories: maldevelopments, predominant white matter injury, predominant gray matter injury, miscellaneous, and normal findings.

### 2.3 Clinical hand function assessment

The MACS (Mcconnell et al., 2011), the Box and Block Test (BBT) (Liang et al., 2021), and the MMs by Woods and Teuber procedure were carried out by two pediatric physical therapists (BC and EB) who were blind to child functional level and brain lesion severity.

#### 2.3.1 Assessment of gross manual dexterity

BBT is a standardized test for measuring gross manual dexterity that can be used for a wide range of populations, from childhood to adulthood. It is quick to administer, simple and inexpensive; it is composed of a “test box” divided into two compartments by a central partition and 150 wooden blocks (25 mm in size). Subjects are asked to transport the blocks from one compartment to the other as quickly as possible in 1 min. The number of blocks transported from one side to the other is recorded (Bleyenheuft et al., 2015). A higher number of blocks corresponds to a better manual dexterity. The subject is asked to perform the test with the dominant hand first (less affected hand), followed by the non-dominant hand (more affected hand), in our cohort of UCP children.

#### 2.3.2 Assessment of mirror movements

**Figure 1:**
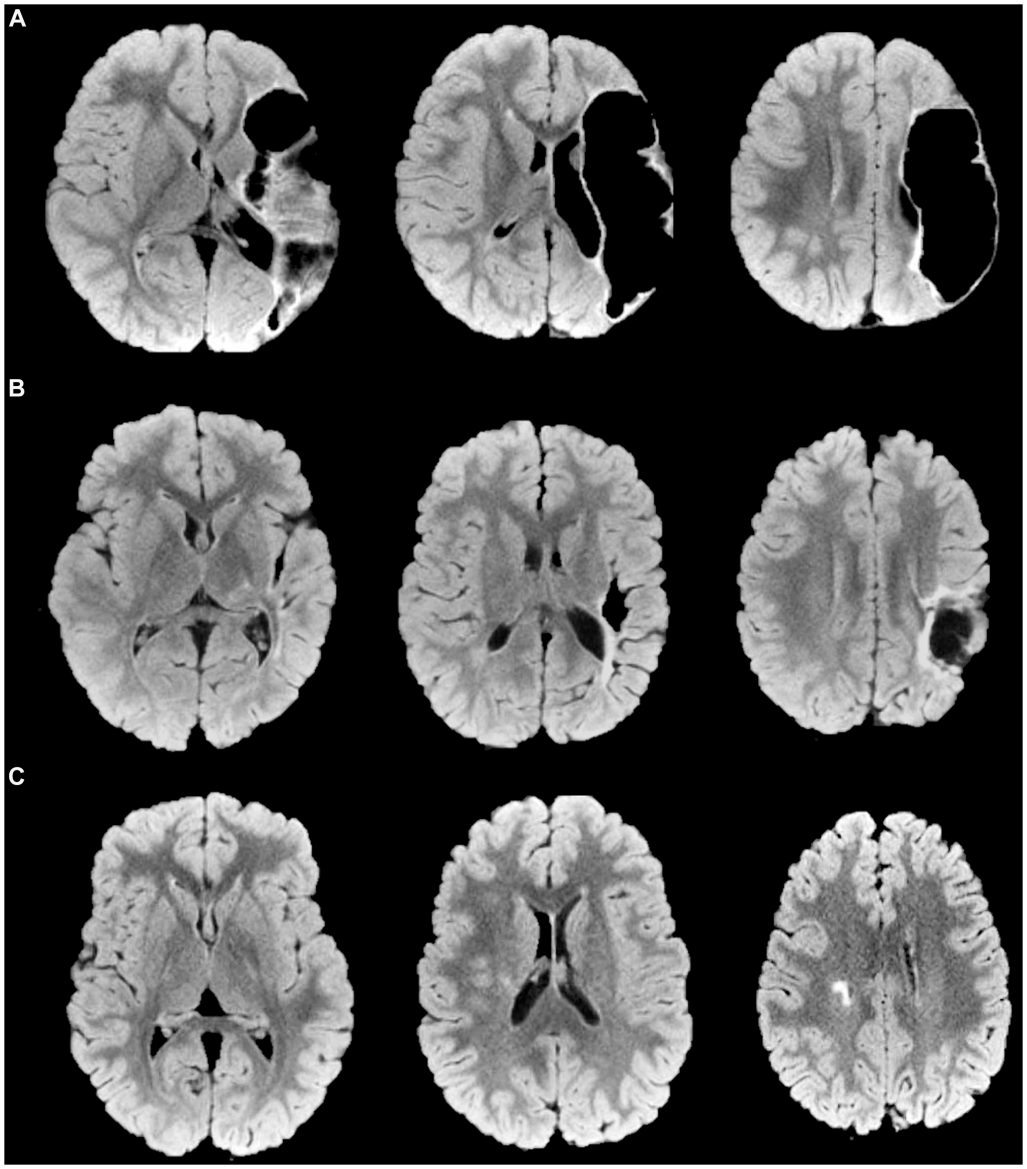
Magnetic Resonance Imaging axial FLuid-Attenuated Inversion Recovery (FLAIR) representative images of some different brain lesion type and severity are provided, with corresponding scores for manual functional impairment and Mirror Movements (MMs). **(A)** Perinatal stroke in the left middle cerebral artery territory (main branch). MRICS type C, predominant gray matter injury. Brain lesion severity score sqMRI GS: 20.5. More affected hand BBT, 13; MMs, 2. Less affected hand BBT, 48; MMs, 7. **(B)** Perinatal stroke in the left MCA territory (cortical branch). MRICS type C, predominant gray matter injury. Brain lesion severity score sqMRI GS, 13.5. More affected hand BBT, 48; MMs, 4. Less affected hand BBT, 13; MMs, 1 **(C)** Right periventricular venous infarction. MRICS B, predominant white matter injury. Brain lesion severity score sqMRI GS, 4. More affected hand BBT, 34; MMs, 2. Less affected hand BBT, 47; MMs, 2. GS, global Score; MRICS, Magnetic Resonance Imaging Classification System; BBT, Box and Block Test; MACS, Manual Ability Classification System; MMs, Mirror Movements.

For the MMs assessment, children are seated comfortably at a table. They are asked to perform three standardized unimanual tasks: (i) fist opening and closing, (ii) finger opposition to the thumb, and (iii) finger tapping (Eyre, 2007). MMs in the opposite hand, while the other hand executed the task are observed, and scored by, using the Woods and Teuber criteria (Woods and Teuber, 1978). Specifically, each task is assigned a score on a four-point scale, with 0 indicating the absence of MMs (“no clear imitative movements”) and 4 indicating symmetrical movements (“movement equal to those observed in the active hand”). According to Woods and Teuber criteria, MMs scores for each hand may range from 0 to 12, with higher scores corresponding to more severe MMs on the observed side. MMs assessment was videotaped with a video camera placed to ensure the best view according to the procedure (Figure 1).

#### 2.3.3 Manual ability classification system

The MACS describes children’s self-initiated ability to manipulate objects and their need to request assistance or adaptation for executing manual activities in everyday life. It ranges from level I, which classify the best level of independence (“handles objects easily and successfully”), to level V (“does not handle objects and has severely limited ability to perform even simple actions”), in which total assistance is needed (Eliasson et al., 2006).

### 2.4 Statistical analyses

Descriptive statistics are reported as mean (SD) or median (range: min-max) according to variables’ distribution. MMs severity was compared between the two hands by using non-parametric Wilcoxon signed-rank test.

Non-parametric partial correlations were applied to assess the associations between brain lesion characteristics at MRI (sqMRI assessment and MRICS category) and clinical measures. For the sqMRI assessment, to evaluate the independent impact of cortical or subcortical lesion load, each cortical and subcortical severity score of the semi-quantitative MRI scale was included separately in the analyses. To the purposes of this study, the HS and BGrS were considered separately for each brain hemisphere; in particular, HS and BGrS contralateral to UCP were included in the analysis and referred to as HSc and BGrSc, respectively. The CCS and GS were also included in the analysis. Finally, MRICS categories (Himmelmann et al., 2017) were also included in the analyses. Clinical upper limb measures included the MACS level, the BBT score and MMs severity score. BBT and MMs were assessed and included in this analysis separately for each hand. The brain lesion laterality (right/left) and participants’ age were included as control variables of no interest. We also investigated the possible relationship between hand function (MACS and BBT) and MMs by means of non-parametric partial correlations adjusted for age and brain lesion laterality. Bonferroni corrections were applied to account for multiple comparisons, and adjusted *p*-values were compared to significance level of α = 0.05. All significant p-values and statistical trend for the correlation analysis are reported after Bonferroni correction.

Moreover, an exploratory regression analysis was run to determine the contribution of different factors to the variability of clinical measures (MACS and BBT) and MMs. Three multiple linear regression models were run with MACS and BBT assessed separately for each hand as dependent variable in their respective models, and participants’ age, brain lesion characteristics (HSc, BGrSc, MRICS and brain lesion laterality) and MMs in each hand as predictors. Two further models were run with MMs in each hand as dependent variables and participants’ age and brain lesion characteristics as predictors. Given the exploratory nature of the regression models, no correction for multiple comparisons was applied in this analysis.

All statistical analyses were run using SPSS v. 26.0.

## 3 Results

### 3.1 Participant characteristics

In total, 35 children and adolescents (20 males, 15 females) aged 4–18 years and diagnosed with UCP were recruited in this study. The age range for brain MRI was 3–18 years. Table 1 summarizes participants’ demographic and clinical characteristics, distribution of MRICS categories and severity of the brain lesion. Significantly more severe MMs resulted in the less affected hand compared to the more affected one (*p* = 0.002).

**Table 1:** Demographic, functional, neurological and neuroanatomical characteristics of patients.

Characteristics		Subjects (*N* = 35)
Age at clinical assessment		9.2 (3.5) years
Age at brain MRI		7.8(4.1) years
Sex	Female	12 (34%)
	Male	23 (66%)
UCP side	left	12 (34%)
	right	23 (66%)
GMFCS	I	32 (91%)
	II	3 (9%)
MACS	I	11 (31%)
	II	13 (37%)
	III	11 (32%)
BBT	Less affected hand	50.4(13.2)
	More affected hand	23.9(14.5)
MMs	Less affected hand	5(0–10)
	More affected hand	3(0–8)
MRICS	I	1 (3%)
	II	14 (40%)
	III	20 (57%)
Semi-quantitative MRI (sqMRI) scoring system	HSc	5.5(0–11.5)
	BGrSc	4(0–8)
	CCS	2(0–3)
	GS	13(3–23)

Items in italics are expressed as mean (SD) or median (range min-max) according to variables’ distribution. UCP, unilateral cerebral palsy; GMFCS, Gross-Motor Function Classification System; MACS, Manual Ability Classification System; BBT, Box and Block Test; MMs, Mirror Movements; MRICS, Magnetic Resonance Imaging Classification System; MRI, Magnetic Resonance Imaging; HSc, Hemispheric Score contralateral to the more affected side; BGrSc, Basal Ganglia region Score contralateral to more affected side; CCS, Corpus Callosum Score; GS, Global Score.

Thirty-two subjects in our cohort had pure unilateral brain injury. Three subjects had bilateral injury with the median HS in the hemisphere ipsilateral to the more affected hand being 0.5 (range min–max, 0.5–4) at the sqMRI assessment, and no subcortical involvement (BGrS = 0).

### 3.2 Associations between brain lesion severity on sqMRI and upper limb function, dexterity and MMs

Results from correlation analyses revealed that brain lesion severity scores were moderately to strongly (Rho range: 0.414–0.624) (Dancey and Reidy, 2007) correlated with upper limb motor function measures. Detailed results of these analyses are reported in Table 2. Overall, more severe brain lesions were associated with worse function at all clinical measures. In particular, after Bonferroni correction, HSc moderately correlated with MACS (Rho = 0.445; *p* = 0.025), BBT in the more affected hand (Rho = −0.434; *p* = 0.030) and less affected hand (Rho = −0.414; *p* = 0.040). BGrSc moderately correlated with MACS (Rho = 0.542; *p* = 0.001) and BBT in the more affected hand (Rho = −0.587; *p* < 0.001). CCS showed a moderate correlation with BBT in the more affected hand (Rho = −0.542; *p* = 0.005), while a trend toward significance emerged with MACS (Rho = 0.385 *p* = 0.065). A strong correlation was found between GS and MACS (Rho = 0.624; *p* < 0.001), and between GS and BBT in the more affected hand (Rho = −0.619; *p* < 0.001), while a trend emerged between GS and BBT in the less affected hand (Rho = −0.394 *p* = 0.060). No correlations were found between sqMRI and MMs.

**Table 2:** Non-parametric partial correlation between brain structure and hand function results controlled for age and brain lesion laterality.

			More affected hand		Less affected hand	
		MACS	BBT	MMs	BBT	MMs
Hemispheric Score* (sqMRI HS)	Rho (*p* value)	**0.445** (0.025)	**−0.434** (0.030)	0.014 n.s.	**−0.414** (0.040)	0.030 n.s.
Basal Ganglia region Score* (sqMRI BGrS)	Rho (p value)	**0.542** (0.001)	**−0.587** (<0.001)	−0.097 n.s.	−0.269 n.s.	0.033 n.s.
Corpus Callosum Score (sqMRI CCS)	Rho (*p* value)	0.385 (0.065)	**−0.542** (0.005)	0.142 n.s.	−0.324 n.s.	0.166 n.s.
Global Score (sqMRI GS)	Rho (*p* value)	**0.624** (<0.001)	**−0.619** (<0.001)	0.054 n.s.	−0.394 (0.060)	0.136 n.s.
MRICS	Rho (*p* value)	0.090 n.s.	−0.055 n.s.	**−0.568** (0.005)	−0.229 n.s.	−0.401 n.s.

sqMRI, semi-quantitative Magnetic Resonance Imaging; MRICS, Magnetic Resonance Imaging Classification System; BBT, Box and Block Test; MACS, Manual Ability Classification System; MMs, Mirror Movements; Rho, Correlation Coefficient. Significant effects and statistical trends are reported after Bonferroni correction for multiple comparisons. *Contralateral to the more affected side.

### 3.3 Associations between MRICS and upper limb function, dexterity and MMs

No association emerged between MRICS category and BBT in either hand. A moderate correlation was found between MRICS and MMs in the more affected hand (Rho = −0.568; *p* = 0.005), with presumed earlier lesions being related with more severe MMs.

### 3.4 Associations among hand function, dexterity and MMs

Regarding clinical measures, MACS showed a strong correlation with BBT in the more affected hand (Rho = −0.828, *p* < 0.001), with higher MACS levels (corresponding to a worse function) being associated with a reduced dexterity in the more affected hand. No associations emerged between MACS and BBT in the less affected hand. After the Bonferroni correction, a trend toward significance indicated a relationship between MMs in the more affected hand and MACS (Rho = 0.378, *p* = 0.078), with more MMs being associated to a higher MACS level. Also, a trend toward significance showed a relationship between MMs and BBT in the less affected hand (Rho = −0.377, *p* = 0.078), with more MMs in the less affected hand being associated with reduced dexterity. The correlation analysis for clinical and neurological measures revealed no further significant association.

### 3.5 Neuroanatomical factors impacting hand function

The exploratory linear regression analyses revealed overall significant models, mostly with a moderate strength of the relationship between the predictors, including neuroanatomical and developmental (age) factors, and the dependent variable (MACS, BBT and MMs). Detailed results are reported in Table 3. Briefly, the model predicting MACS showed R2 = 0.57, *p* = 0.006. Individual predictors examination indicated that only BGrSc showed an independent significant contribution to MACS variability. The model predicting BBT in the more affected hand showed R2 = 0.60, *p* = 0.004. Individual predictors examination indicated that BGrSc showed significant contribution to BBT variability, while age reached a trend toward statistical significance. The model predicting BBT in the less affected hand showed R2 = 0.47, *p* = 0.041. In this model, only age showed an independent significant contribution to BBT variability.

**Table 3:** Detailed results of the linear regression models for brain structure, age and hand function.

				More affected hand				Less affected hand			
		MACS		BBT		MMs		MACS		BBT	
Model summary	R^2^ (*p* value)	0.57(0.006)		0.60(0.007)		0.43(0.017)		0.47(0.041)		0.36(0.051)	
	F	3.99		4.41		3.48		2.62		2.62	
		Beta (CI95%)	*p*	Beta (CI95%)	*p*	Beta (CI95%)	*p*	Beta (CI95%)	*p*	Beta (CI95%)	*p*
Predictors	Hemispheric Score* (sqMRI HS)	0.17(−1.5,2.8)	n.s.	−1.11(−2.0,1.1)	n.s.	0.34(−0.3,0.5)	0.081	0.01(−1.6,1.9)	n.s.	0.28(−0.1,0.6)	n.s.
	Basal Ganglia region Score* (sqMRI BGrS)	**0.57(0.1,0.3)**	**0.003**	**−0.48(−4.3,-0.7)**	**0.007**	0.03(−0.3,0.4)	n.s.	−0.06(−2.4,1.8)	n.s.	0.05(−0.4,0.5)	n.s.
	MRICS	−0.09(−0.7,0.5)	n.s.	0.22(−4.6,14.5)	n.s.	**−0.67(−3.8,-1.1)**	**0.001**	−0.18(−15.7,7.1)	n.s.	**−0.49(−3.9,-0.4)**	**0.017**
	Lesion laterality	0.17(−0.2,0.7)	n.s.	0.00(−7.8,7.8)	n.s	0.09(−1.0,1.8)	n.s.	−0.02(−9.8,8.9)	n.s.	0.07(−1.5,2.2)	n.s.
	Age	0.001(−0.1,0.1)	n.s.	0.31(−0.2,2.5)	0.090	−0.32(−0.4,0.2)	0.081	**0.60(0.7,3.9)**	**0.007**	**−0.44(−0.6,-0.5)**	**0.024**
	MMs More affected hand	0.24(−0.1,0.2)	n.s.	−0.09(−3.1,1.9)	n.s	**–**		−0.07(−3.5,2.6)	n.s.	–	
	MMs Less affected hand	0.04(−0.1,0.1)	n.s.	−0.17(−2.8,1.1)	n.s.	**–**		−0.01(−2.4,2.3)	n.s.	–	

MACS, Manual Ability Classification System; BBT, Box and Block Test; MMs, Mirror Movements; sqMRI, semi-quantitative MRI; MRICS, Magnetic Resonance Imaging Classification System. *Contralateral to the more affected side.

The model predicting MMs in the more affected hand showed R2 = 0.43, *p* = 0.017. Individual predictors examination indicated that MRICS showed a significant independent contribution to MMs variability, while HSc and age reached a trend toward statistical significance. The model predicting MMs in the less affected hand approximated statistical significance by showing an R2 = 0.36, with *p* = 0.051. In this model, MRICS and age showed independent significant contribution to MMs variability.

## 4 Discussion

Brain lesion severity demonstrated a relationship with UL functional impairment in children with UCP. The sqMRI scores contralateral to the more affected hand and GS moderately correlated with measures of gross manual dexterity (BBT) and manual ability (MACS) in the more affected hand. As cortical (HSc) and subcortical (BGrSc) structures have been assessed separately, these results support the contribution of both cortical and subcortical brain structures to UL functional impairment in UCP. According to previous literature (Dinomais et al., 2016) the corpus callosum has a potential independent impact on functional impairment and neurological characteristics in UCP; therefore, it was included as a separate score in the analyses. Results revealed that the more severe the corpus callosum involvement by brain injury, the more impaired gross manual dexterity and possibly manual ability in the more affected hand. Overall, these findings are consistent with previous literature showing brain lesion severity assessed on clinical MRI being related to functional impairment (Levitt, 1983; Dinomais et al., 2016). No association was found between MRICS categories and UL functional impairment in our cohort.

A previous study reported relationships between brain lesion severity and upper limb motor function, but using different clinical measures than those used in the current study (Krumlinde-sundholm et al., 2003). These included the Assisting Hand Assessment (AHA), which explores the ability of the impaired hand as an assisting hand in bimanual tasks (Krumlinde-sundholm et al., 2003); the Melbourne Assessment of Unilateral Upper Limb Function (MUUL) that measures unimanual capacity of the impaired hand (Johnson et al., 1994), and the Jebsen–Taylor Test of Hand Function (JTHFT) that assesses speed and dexterity of the impaired upper limb (Jebsen et al., 1969). Compared to previous findings, current results demonstrate associations among several brain lesion severity scores in the hemisphere contralateral to the more affected hand, gross manual dexterity at BBT and hand function in daily activities at MACS. BBT has previously showed reliability and validity against several clinical measures in UCP (Liang et al., 2021); results from the current study further support its construct validity versus a measure of brain lesion severity at clinical MRI (Portney and Watkins, 2009). Moreover, and in agreement with previous findings (Decraene et al., 2021; Liang et al., 2021), results from the current study support the use of BBT in the clinical and research setting, as it is a user-friendly, easily accessible, and simple to administer test, that does not require specialized settings and is valid toward clinical and brain neuroanatomical features in UCP. Brain structure-hand function correlation analyses also showed that both gross manual dexterity at BBT in the more affected hand and manual ability at MACS correlated with sqMRI scores. Furthermore, a strong correlation emerged between BBT in the more affected hand and MACS in our cohort of subjects with UCP. Overall, such results are in line with previous reports that explored the relationship between manual ability and gross dexterity (Krumlinde-Sundholm and Eliasson, 2002; Golubović and Slavković, 2014). In addition, the association between BBT more affected side and MACS, previously reported as moderate in CP (Zapata-Figueroa and Ortiz-Corredor, 2022), was strong in our group of UCP children, likely due to the more homogeneous nature and distribution of their motor disorder in the UL. Finally, no association emerged between gross manual dexterity in the less affected hand and MACS, likely supporting the prominent role of the more affected hand functional impairment in manual ability of children with UCP.

There is a growing evidence in support of a decrease in the dominant hand’s motor performance in children with UCP as compared to typically developing children (TDC) (Woods and Teuber, 1978; Gordon and Duff, 1999; Fiori et al., 2015). It was also recently demonstrated that manual dexterity and rate of hand fine motor skill development in the less affected hand of UCP children are markedly lower than those of TDC (Koerte et al., 2010). To this purpose, we calculated post-hoc the percentage of subjects in our sample with gross manual dexterity at BBT below age-expected performance (Laporta-Hoyos et al., 2018) in the less affected hand. Indeed, this analysis showed that 17% (7 out of 35) of UCP children in our cohort have impaired gross unimanual dexterity in the less affected side, which is not negligible, and further support existing literature (Burn and Gogola, 2022). Neuroanatomical correlates of manual dexterity of the less affected hand in UCP remain to be fully elucidated. Interestingly, our results show that only the HSc has a moderate association with gross manual dexterity in the less affected hand, with a trend toward significance for the GS. Bilateral asymmetrical brain injury may be first hypothesized to be responsible for this functional impairment. However, only one subject among those with impairment of the less affected hand in our cohort has bilateral (largely asymmetrical) brain injury at clinical MRI. Our findings, instead, seem to support the hypothesis of a possible impact of brain lesion severity in the hemisphere contralateral to the more affected side on functional impairment in the ipsilateral hand. As subcortical brain lesion severity did not show any relationship with gross manual dexterity, we can speculate that cortical aspects of brain injury may have a prominent role in hand functional impairment of the less affected hand in comparison to the subcortical lesion load. It is worth noting that the use of clinical MRI cannot exclude the presence of some lesion-induced microstructural or functional brain abnormalities that may be responsible for the less affected side impairment. Quantitative advanced neuroimaging techniques may however reveal such abnormalities and further explain aspects of hand functional impairment that cannot be depicted by conventional brain MRI (Fiori et al., 2015; Scheck et al., 2016; Pagnozzi et al., 2020).

In our cohort, significantly more severe MMs were observed in the less affected hand. Brain lesion severity showed no association with MMs, in either hand. Conversely, children with MRICS category corresponding to presumed earlier brain injury showed more MMs in the more affected hand, while no association emerged with the other hand. These findings are aligned with previous literature in UCP (Carr et al., 1993; Nelles et al., 1998; Kuhtz-Buschbeck et al., 2000; Klingels et al., 2016).

With regards to the association between MMs and functional impairment, our results showed only trends for statistical significance after correction for multiple comparisons. According to this, more severe MMs in the more affected hand were possibly associated to a worse MACS level. As it is, this finding differs from previous literature that fails in finding associations between manual ability in the more affected hand and homolateral MMs (Nelles et al., 1998). Also, our results showed that the presence to more severe MMs possibly corresponded to a higher impairment in homolateral gross manual dexterity in the less affected hand. Since it was previously hypothesized that MMs in this hand are a non-specific motor phenomenon, due to the repeated effort of the more affected hand toward voluntary movement, it can be speculated that this association might be related to maladaptive compensation. However, further studies are warranted to better clarify these associations.

In order to explore the contribution of neuroanatomical characteristics of brain injury (MRICS category and severity of brain insult), MMs and developmental (age) factors to UL functional impairment in UCP, multiple regression model analyses were performed. The manual ability in daily life at MACS was largely explained (57% explained variability) by included predictors. Brain lesion severity in the subcortical region (BGrSc) of the hemisphere contralateral to the more affected side was the main factor impacting on MACS. This finding supports our initial hypothesis on the relationship between brain lesion characteristics and variability of functional impairment. However, since the sqMRI scale BGrS includes the basal ganglia assessment as well as the assessment of the thalamus, the posterior limb of internal capsule and brainstem, in this study it is not possible to disentangle the specific contribution of each of these structures (De Vries et al., 1999; Tsao et al., 2014; Weinstein et al., 2014). Further studies are mandatory to better identify the specific role of pyramidal and extrapyramidal structures to hand moto control.

The gross manual dexterity in the more affected hand was largely explained by the model including neuroanatomical factors (MRICS category and severity of brain insult), MMs and age (60% explained variability). In particular, manual dexterity was highly impacted by brain lesion severity in the subcortical region and, to a less extent, by age. This is fully in agreement with physiological processes of maturation of manual dexterity over developmental ages (Laporta-Hoyos et al., 2018). Furthermore, in our results, the only predictor of the gross manual dexterity in the less affected hand was age (model explained variability of 47%), while no impact of brain lesion severity emerged.

We finally explored the variability of UL MMs in either hand according to neuroanatomical (MRICS category and severity of brain insult) and developmental factors (age). Both models explained a certain amount of variability of MMs (43 and 36%, respectively, for the more and the less affected hand). Interestingly, factors impacting UL MMs in the more affected hand were the MRICS category of brain injury, and to a lesser extent, the severity of brain injury in cortical hemispheres (but not subcortical structures) and age. The model explaining UL MMs in the less affected hand only approximated significance in our study. Age and only the MRICS category, among the neuroanatomical factors, significantly accounted for explained variability. These differences in significance levels and predictors between MMs in the more affected hand compared to the less affected one might support previous literature that identifies MMs in the more affected hand as more directly related to aspects of brain injury, while other factors may contribute to MMs genesis and severity on the less affected side in UCP (Nelles et al., 1998; Staudt et al., 2004). Specifically, MMs in the more affected hand are usually considered an epiphenomenon of CST reorganization, and their severity could be hypothesized to be related to characteristics of cortical brain lesion (Klingels et al., 2016). Results from the present study align with prior findings. However, further studies involving larger cohorts and using more advanced structural and functional neuroimaging and electrophysiological techniques are mandatory to validate this hypothesis. Finally, according to this perspective, the higher presence of MMs in the less affected hand, both in our and previous studies, may instead reflect the contribution of maladaptive synkinesis phenomena that are not substantially related to brain lesion characteristics.

This study has limitations. Age range is wide in our cohort of subjects with CP. Due to the impact of age on our measures, our data analyses were adjusted for age; further studies limited to more homogeneous age-ranges may better clarify the robustness of results over years during developmental ages. Also, we did not account for quality and quantity of weekly habilitative programs our subjects might be part of, despite the fact that we excluded subjects that performed intensive UL trainings any time before enrolment. This factor should therefore be evaluated in future investigations. Brain MRI in our cohort was performed asynchronously with respect to functional clinical assessment; however, since myelination has been described to reach an adulthood signal pattern at brain MRI after 2 years of age (Welker and Patton, 2012), we do not expect any significant bias due to incomplete myelination in sqMRI brain lesion severity assessment in our cohort. Furthermore, although clinical imaging has several advantages in terms of availability and feasibility in children, more advanced neuroimaging techniques may support a deepen exploration of such complex processes compared to brain function and plasticity that, instead, can only be hypothesized by clinical approaches. Also, quantitative kinematic analyses are available for a detailed quantitative definition of functional impairment in UCP. No data concerning body mass index or daily activities for our CP subjects were included in the current study. These may have an impact on generalization of our findings. Finally, plasticity of the corticospinal system was only hypothesized based on clinical MMs assessments, given the lack of a TMS-based exact representation of voluntary motor control reorganization.

## 5 Conclusion

Based on clinical easily accessible measures, our results support the importance of brain lesion neuroanatomical characteristics to understand and explain variability of UL functional impairment in UCP. The presence of relationships among brain lesion severity, gross manual dexterity of the more affected hand, and manual ability in UCP is consistent with previous literature. Our results further support the validity of the sqMRI assessment to understand aspects of brain structure–function relationship and to be used for clinical and research purposes in contexts with limited resources, where advanced imaging is not accessible. Finally, the sqMRI approach and BBT mutually reinforce their value as clinical measures to be applied in the research setting for studies on UL functional impairment in UCP.

## Data availability statement

The original contributions presented in the study are included in the article/supplementary material, further inquiries can be directed to the corresponding author.

## Ethics statement

The studies involving humans were approved by Pediatric Ethics Committee of Tuscany. The studies were conducted in accordance with the local legislation and institutional requirements. Written informed consent for participation in this study was provided by the participants’ legal guardians/next of kin. Written informed consent was obtained from the individual(s), and minor(s)’ legal guardian/next of kin, for the publication of any potentially identifiable images or data included in this article.

## Author contributions

EB: Conceptualization, Data curation, Formal analysis, Investigation, Methodology, Supervision, Writing – review & editing, Writing – original draft. VB: Methodology, Writing – original draft, Writing – review & editing. ES: Data curation, Methodology, Writing – review & editing. BC: Data curation, Methodology, Writing – review & editing. FF: Data curation, Formal analysis, Methodology, Writing – review & editing. SFil: Data curation, Methodology, Writing – review & editing. GC: Conceptualization, Supervision, Writing – review & editing. SFio: Conceptualization, Data curation, Formal analysis, Investigation, Methodology, Supervision, Writing – review & editing. GS: Conceptualization, Data curation, Funding acquisition, Investigation, Methodology, Project administration, Resources, Supervision, Writing – review & editing.
